# Molecular evidence of functional progesterone withdrawal in human myometrium

**DOI:** 10.1038/ncomms11565

**Published:** 2016-05-25

**Authors:** Lubna Nadeem, Oksana Shynlova, Elzbieta Matysiak-Zablocki, Sam Mesiano, Xuesen Dong, Stephen Lye

**Affiliations:** 1 Lunenfeld Tanenbaum Research Institute, Mount Sinai Hospital, Toronto, Ontario, Canada M5T 3H7; 2 Department of Obstetrics & Gynecology, University of Toronto, Toronto, Ontario, Canada M5G 1E2; 3 Department of Reproductive Biology, Case Western Reserve University, Cleveland, Ohio 44106-5034, USA; 4 Department of Urologic Sciences, Vancouver Prostate Centre, University of BC, Vancouver, British Columbia, Canada V6H 3Z6; 5 Department of Physiology University of Toronto, Toronto, Ontario, Canada M5S 1A1

## Abstract

Progesterone suppresses uterine contractility acting through its receptors (PRA/B). The mechanism by which human labour is initiated in the presence of elevated circulating progesterone has remained an enigma since Csapo first theorized of a functional withdrawal of progesterone in 1965. Here we report that *in vitro* progesterone-liganded nuclear PRB forms a complex including JUN/JUN homodimers and P54^nrb^/Sin3A/HDAC to repress transcription of the key labour gene, *Cx43*. In contrast, unliganded PRA paradoxically activates Cx43 transcription by interacting with FRA2/JUND heterodimers. Furthermore, we find that while nuclear progesterone receptor (PR) is liganded during human pregnancy, it becomes unliganded during both term and preterm labour as a result of increased expression of the progesterone-metabolizing enzyme 20α HSD and reduced nuclear progesterone levels. Our data provide a mechanism by which human labour can occur in the presence of elevated circulating progesterone and suggests non-metabolizable progestogen might represent an alternative new therapeutic approach to preterm birth prevention.

## Abstract (web summary)

How human labour is initiated in the presence of high circulating progesterone is still unclear. Here, the authors show that during a reduction in myometrial nuclear progesterone levels, there is an increased transcription of the key labour gene, Cx43, mediated by unliganded progesterone receptor A.

## Graphical abstract

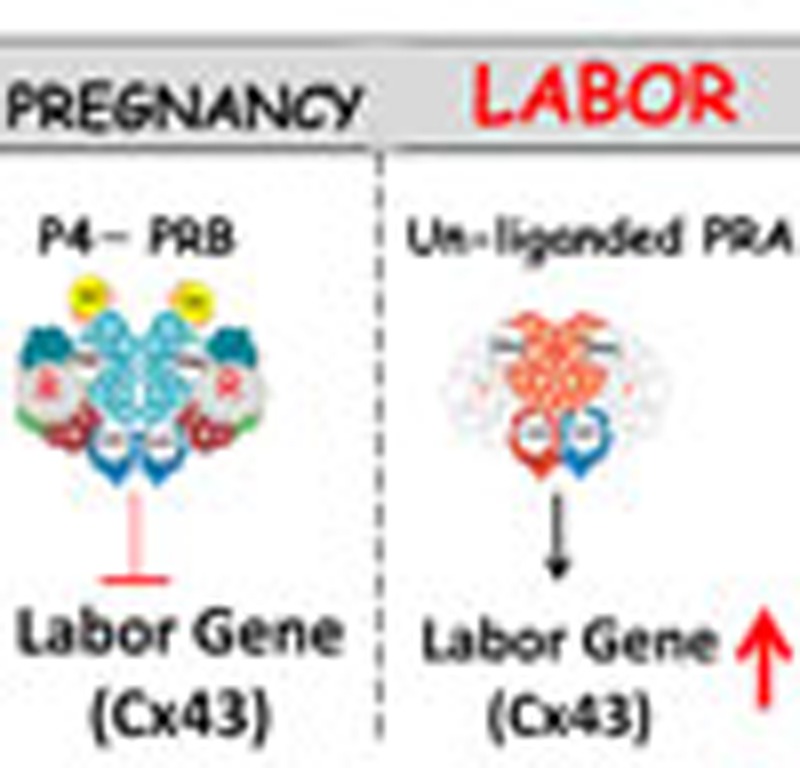

More than 85 years after the discovery of progesterone (P4) by Corner and Allen^1^, we have limited understanding of its molecular role in pregnancy maintenance and the initiation of labour. Progesterone suppresses spontaneous uterine contractility during pregnancy and, in most mammals, a fall in systemic P4 levels (‘progesterone withdrawal') is required for the initiation of labour at term. However, in humans, labour occurs in the presence of elevated circulating levels of P4, leading Csapo to propose the ‘functional P4 withdrawal' theory^2^,^3^ though the mechanisms by which this is achieved have yet to be defined.

Over many decades researchers have proposed hypotheses to explain the functional withdrawal of P4, including, the sequestration of active P4 by corticosteroid-binding globulin^4^, a decrease in active P4 metabolite levels^5^, changes in the ratio of progesterone receptor (PR) isoforms^6^ or transcriptional co-activators and/or repressors^7^, a functional oestrogen activation^8^ and inflammation resulting in NF-κB-mediated PR repression^9^. While there is a lack of compelling evidence to support any of these hypotheses, the theory of a ‘functional P4 withdrawal' remains valid since disruption of P4 signalling by the PR antagonist RU486 at any stage of pregnancy results in myometrial contractions and labour in mice^10^, rats^11^ and women^12^, while the treatment of pregnant rodents with P4 delays myometrial contractions and parturition^13^.

One of the hypotheses postulates that the differential expression of the progesterone receptor isoforms, PRA and PRB contributes to a functional withdrawal of P4 and the onset of labour^6^. PRB is the full-length receptor while PRA lacks the 164 amino acid AF3-activation domain at the N terminus^14^,^15^. We have suggested that PRB dominates throughout pregnancy and mediates myometrial quiescence, while during labour an increase in the PRA:PRB ratio leads to the functional suppression of PRB and the induction of labour^6^,^16^,^17^. However, until now, the specific mechanisms by which these isoforms contribute to labour onset remain unclear.

Labour requires increased expression of genes (for example, *GJA1*(Cx43), *PTGS2*(COX2), *OXTR* and *NFKB2*) that mediate myometrial ‘activation' and optimal responsiveness to uterotonic agonists such as stimulatory prostaglandins and oxytocin. Among these labour genes, the gap junction protein Cx43 plays an essential role in the initiation of term or preterm labour through cell–cell coupling and generation of synchronous myometrial contractions^18^,^19^,^20^,^21^,^22^,^23^. The *Cx43* gene is regulated by the AP-1 (Fos/Jun) family of transcription factors that function as either Jun/Jun homodimers or Fos/Jun heterodimers^24^,^25^. We have shown that Fos/Jun heterodimers are strong inducers of Cx43 transcription compared with Jun/Jun homodimers^26^,^27^ and that P4, acting through PRs, represses Cx43 transcription. The physical interaction between PRs and cJun results in the recruitment of PR-p54^nrb^/mSin3A/HDAC transcriptional repressor complex to the AP-1 consensus site in the Cx43 promoter^28^,^29^.

We now demonstrate that during pregnancy progesterone confers transcriptional repression of myometrial Cx43 through the formation of PRB-JUN/JUN homodimer complex, whereas during labour an increased expression of FOS proteins favours binding of PRA to the Cx43 promoter. Remarkably, during labour nuclear PRA is unliganded, even in the presence of elevated circulating P4 levels and that in this unliganded state, PRA acts as a transcriptional activator (rather than repressor) of Cx43. Finally, we provide evidence that the loss of binding of P4 to PRA is due to a reduction in intracellular P4 levels in the myometrium as a result of increased expression of the metabolizing enzyme, 20α hydroxysteroid dehydrogenase (20αHSD). Altogether, these data provide a mechanistic basis for the functional withdrawal of P4 in human labour.

## Results

### PR differentially interact with AP-1 transcription factors

**Figure 1:**
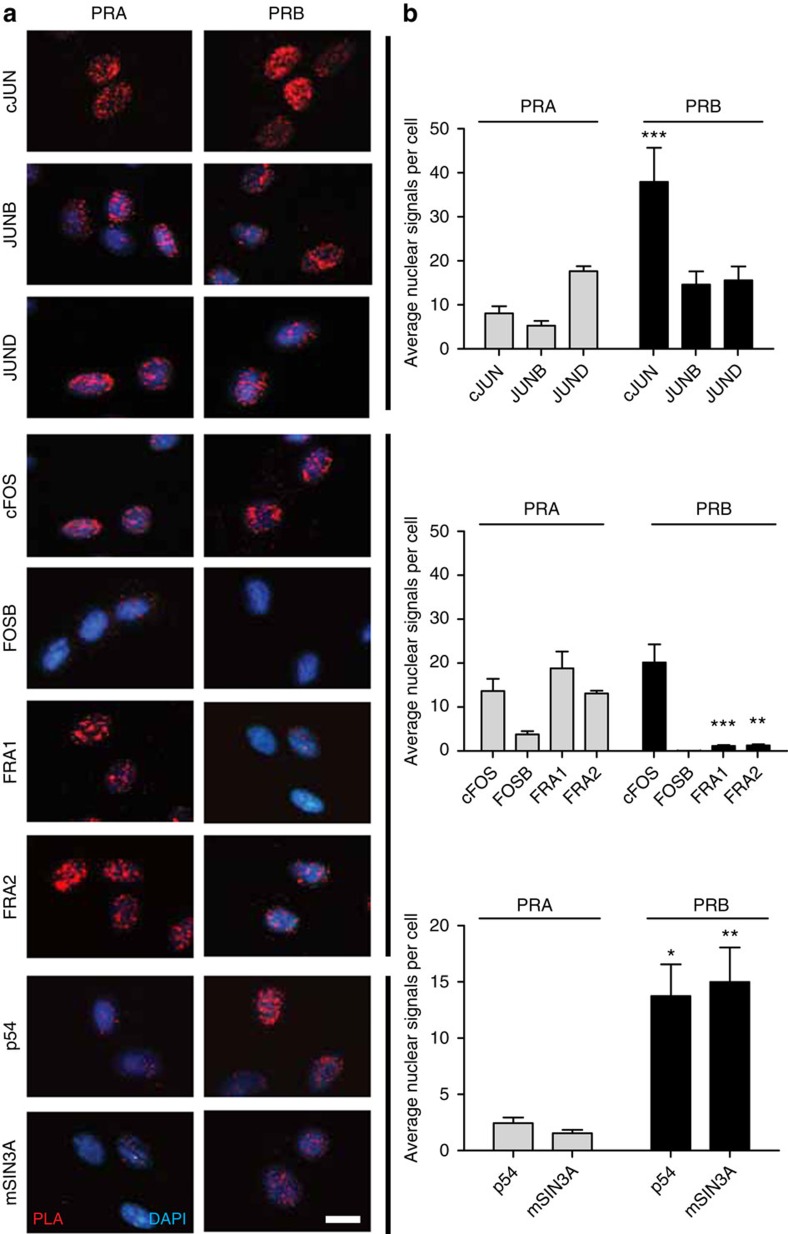
PRA/PRB show differential interaction with AP-1 transcription factors and transcriptional co-repressor proteins p54^nrb^/mSIN3A. *In situ* proximity ligation assay (PLA) of PRs with Jun and Fos family members and co-repressor proteins; p54^nrb^ and mSIN3A. PRA and PRB stable transfected hTERT-HM cells were treated with 100 nM progesterone (P4) for 2 h and then subjected to PLA. (**a**) Representative pictures and (**b**) signal analysis (average nuclear signals/cell from three fields), pooled from three independent experiments are shown. Two-way analysis of variance followed by bonferroni post-tests show significant differences between PRA and PRB in the interaction of cJUN *(P*<0.001, denoted as ***), Fra1 *(P*<0.001), Fra2 (*P*<0.01, denoted as **), p54 *(P*<0.05, denoted as *), and mSIN3A *(P*<0.01). Data represents mean±s.d., *n*=3. Scale bar, 20 μm.

We previously showed that AP-1 proteins modulate transcription of Cx43 (ref. 26). Since an increase in the PRA:PRB ratio has been suggested to mediate a functional withdrawal of P4, we investigated the interaction of PR isoforms with the AP-1 proteins. To differentiate between two PR isoforms, we used human myometrial hTERT-HM cell lines stably expressing Flag-tagged PRA and PRB as described earlier^30^. The endogenous expression of AP-1 proteins in this cell line (Supplementary Fig. 1) and human myometrium (labouring and non-labouring) is shown in Supplementary Fig 2. hTERT-HM cells were stimulated with P4 and subjected to PLA analysis to characterize interactions between AP-1 proteins and PR isoforms (Fig. 1a). Compared with PRA, PRB showed stronger interaction with cJun and JunB proteins, similar affinity towards JunD and cFos and weaker interaction with Fra1 or Fra2 proteins; both receptors exhibited minimal interaction with FosB (Fig. 1a). Quantitation of PLA indicated that there was a significant increase (*P*<0.05) in the affinity of PRA (relative to PRB) to Fra1, Fra2 proteins in a ligand-independent manner (Fig. 1b, Supplementary Fig. 3). We confirmed these interactions using *in vitro* co-immunoprecipitation assay in Syrian hamster myometrial (SHM) cells (Supplementary Fig. 4).

Next, we examined the interaction between PRA/B and the co-repressor proteins p54^nrb^/mSIN3A which mediate Cx43 suppression. PRB showed strong affinity for both co-repressors, whereas PRA had minimal interaction with p54^nrb^ or mSIN3A (Fig. 1a,b).

### AP-1 dimers determine PR modulation of cx43 transcription

**Figure 2:**
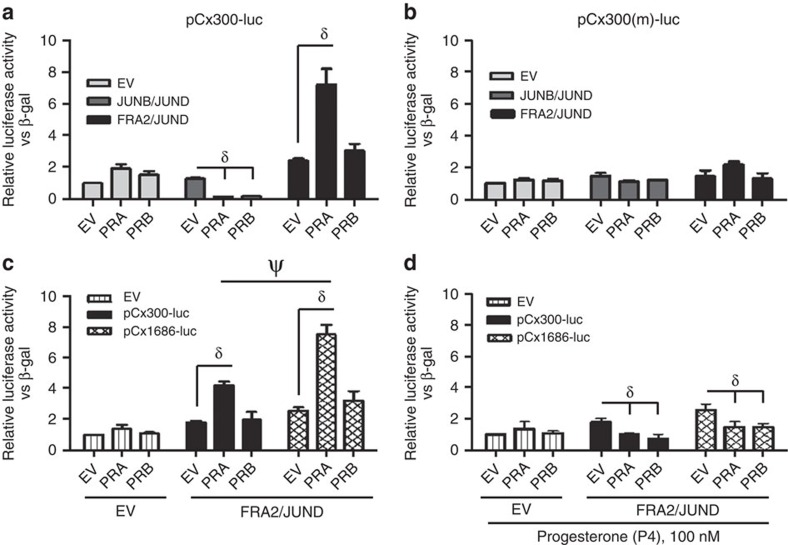
Unliganded PRA activates Cx43 transcription in the presence of Fos/Jun heterodimer. SHM cells were transiently transfected with pCx300-luc, pRSVbgal, PRA or PRB, and (1:1) combination of Jun/Jun or Fos/Jun expression vectors for 48 h. The relative luciferase to β-galactosidase activity is represented as the fold induction over cells transfected with the empty vector control (EV of the EV group). (**a**) Luciferase promoter activity assay: PRs suppress Cx43 transcription in the presence of Jun/Jun dimers but induces it in the presence of Fos/Jun heterodimer (FRA2/JUND). (**b**) Mutation of AP-1 sequence abolishes PRA mediated transactivation of Cx43 promoter. (**c**) Increasing the number of AP-1 sites enhances the transactivation of Cx43 promoter by PRA in the presence of Fos/Jun heterodimer. (**d**) Addition of progesterone (P4) suppressed Cx43 promoter activity by PRs in the presence of heterodimer. δ represents significant difference (*P*<0.05) with respect to EV control of that group and ψ represents significant difference (*P*<0.05) in the PRA of the respective groups as determined by two-way analysis of variance. Data represents mean±s.d., *n*=4 independent experiments.

We next determined the functional impact of the interactions between AP-1 dimers and PR isoforms on Cx43 transcription using SHM cells co-transfected with a Cx43 luciferase promoter (pCx300-luc containing the proximal AP-1 site, Supplementary Fig. 5)^26^,^31^, PRA or PRB isoforms and either JUNB/JUND homodimers or FRA2/JUND heterodimers (FRA2 is the predominant nuclear FOS protein during labour; Supplementary Fig. 2). Our data indicate that the AP-1 dimer composition determines the modulation of Cx43 transcription by the PR isoforms. Thus, in the presence of JUNB/JUND homodimers both PRA and PRB repress transcription of Cx43, while in the presence of FRA2/JUND heterodimers PRA (but not PRB) activates Cx43 transcription (*P*<0.05; Fig. 2a). A two base-pair mutation of the AP-1 consensus sequence from TGAGTCA to TGAGTtg (pCx300(m)-luc, Supplementary Fig. 5) resulted in the abrogation of PR-JUNB/JUND mediated repression and PRA-FRA2/JUND mediated induction of Cx43 promoter activity (Fig. 2b). The activation of Cx43 transcription by PRA-FRA2/JUND was further enhanced using a Cx43 promoter construct that contained both proximal and distal AP-1 sites (pCx1686-luc, Supplementary Fig. 5, Fig. 2c). Importantly, in the presence of progesterone PRA no longer activated Cx43 transcription (Fig. 2d).

### Myometrial PRs are unliganded during labour

**Figure 3:**
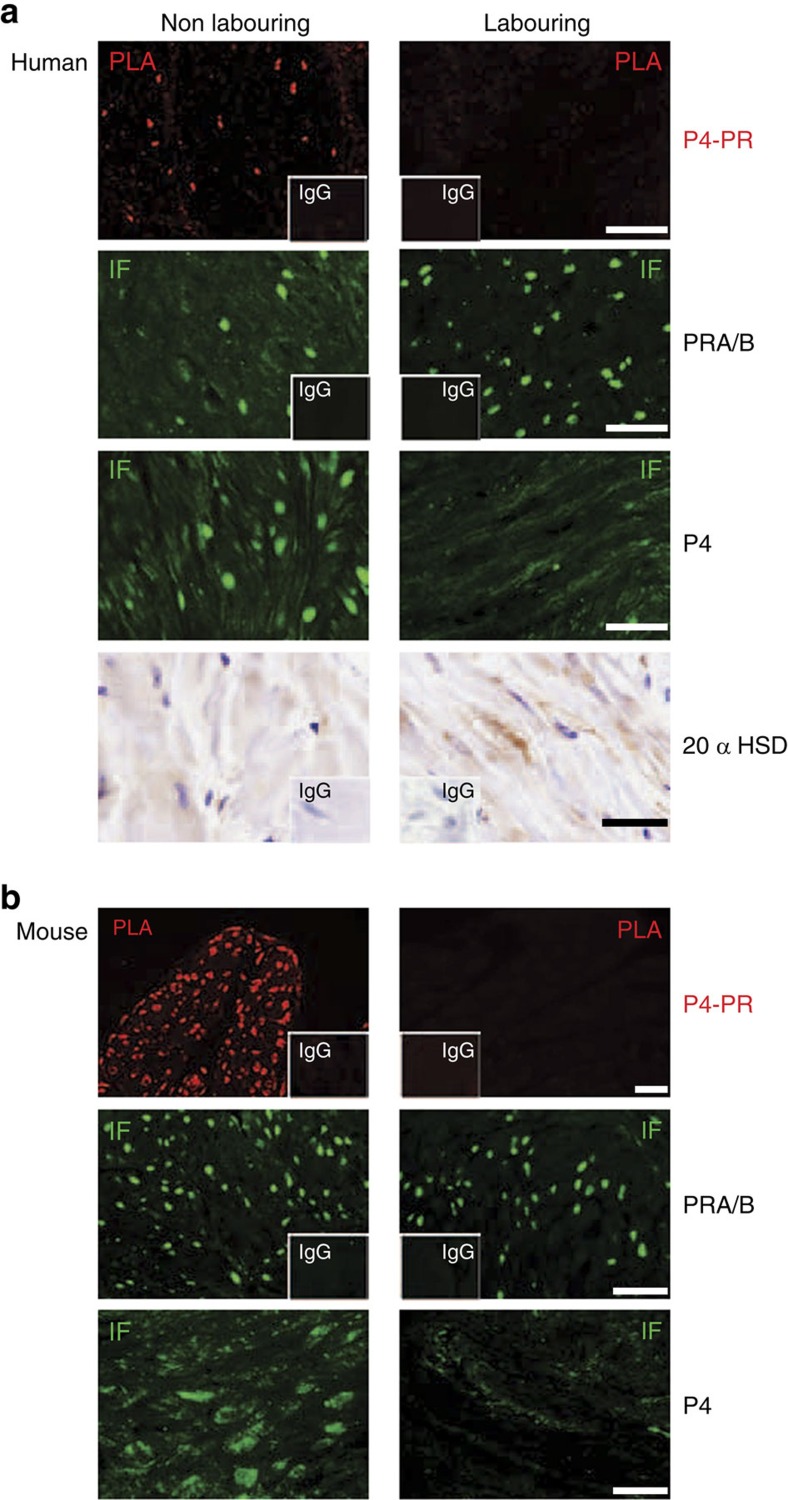
Myometrial PRs are unliganded during labor. (**a**) Term human myometrium from non-labouring and labouring state (*n*=4 per group). Representative pictures of: *in situ* Proximity Ligation Assay (PLA) between PR and P4 where red signal represents P4-liganded PRs, and immunofluorescence (IF) with green signal represents total PRs (PRA/B) and P4, and immunohistochemistry (IHC) for 20α HSD. (**b**) Mouse myometrium from non- labouring and labouring state (*n*=4 per group). IgG controls shown as embedded picture. Scale bar, 50 μm.

Our unexpected *in vitro* finding that unliganded PRA acts as a transcriptional activator of Cx43 led us to investigate whether PRA is unliganded during human labour when Cx43 transcription is greatly increased. Using PLA, we confirmed that before the onset of labour PRs are liganded to P4 in the human myometrium. However, despite the abundance of circulating P4, PRs are unliganded during labour (Fig. 3a). We sought to validate this remarkable observation in an animal model in which a systemic P4 withdrawal leads to the onset of labour. As predicted, PLA demonstrated that PR is liganded during pregnancy when P4 levels are elevated but are unliganded during labour when peripheral and myometrial P4 levels are reduced (Fig. 3b). In both cases the loss of P4–PR binding was not due to a loss of nuclear PR as demonstrated by immunofluorescence.

### Myometrial P4 level is decreased during labour

**Figure 4:**
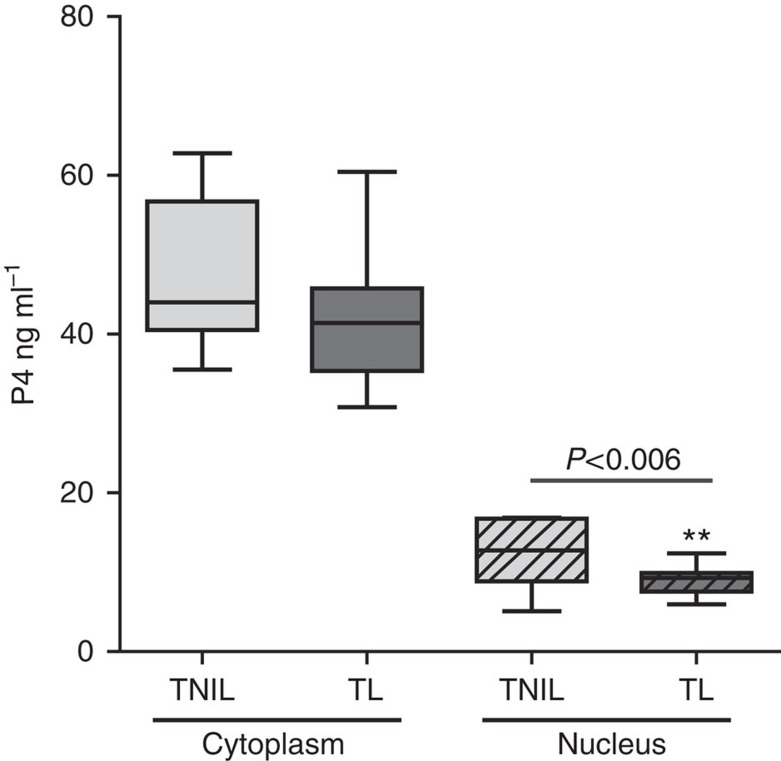
Nuclear P4 levels are decreased in the labouring myometrium. Cytoplasmic and Nuclear tissue lysates were prepared from non-labouring and labouring human myometrium (*n*=11 each). Lysates were subjected to P4 ELISA using DRG Progesterone Enzyme Immunoassay Kit (DRG, Germany). Statistical analysis was performed using *t*-test for equality of variance. Significance was determined at **P<0.01. Data represents mean±s.d.

Next we examined whether the lack of binding of P4 to PR during human labour was due to reduced myometrial P4 levels despite the abundance of circulating P4. Using immunofluorescence, we found that the nuclear P4 levels were decreased in the labouring human myometrium in comparison with non-labouring myometrium (Fig. 3a). Moreover, we found increased expression of the P4-metabolizing enzyme 20α HSD/AKR1C1 in human myometrium during labour compared with non-labouring tissue (Fig. 3a). These data suggest that metabolism of P4 leads to a local withdrawal of the steroid and consequently uncoupling of PR from its ligand. To verify the reduced tissue levels of P4, we conducted ELISA in myometrial tissue lysates from non-labouring and labouring women (*n*=11 per group). We compared cytoplasmic and nuclear P4 levels in human myometrium and found that P4 concentration was three- to fourfold higher in the cytoplasm than in the nucleus. There was no significant difference in the cytoplasmic P4 concentration between labouring and non-labouring human myometrium; however, a significant decrease (*P*<0.006) in the nuclear P4 levels was detected during term labour (Fig. 4).

### Differential localization of liganded and unliganded PRs

**Figure 5:**
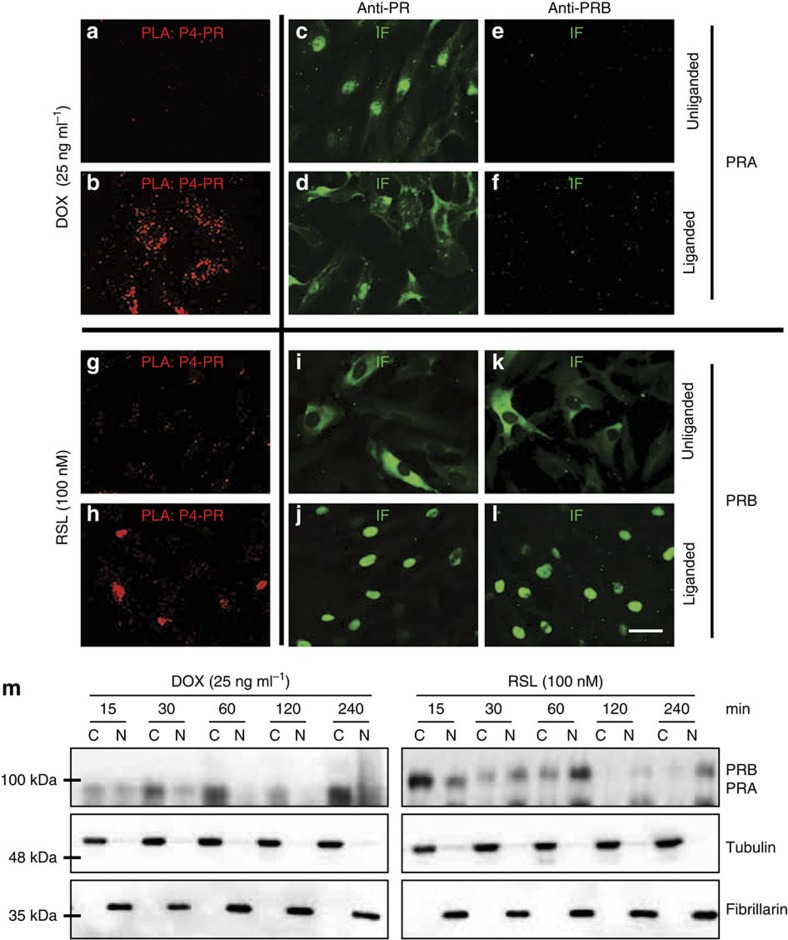
Liganded and unliganded PRA and PRB isoforms have differential cellular distribution. Representative pictures of: *in situ* Proximity Ligation Assay (PLA) and immunofluorescence (IF) for total PRs and PRB in human myometrial cells with inducible PRA (**a**–**f**) and PRB (**g**–**l**) expression system. Cells were induced for 24 h with Dox (25 ng ml^−1^) for PRA and with RSL (100 nM) for PRB expression and then treated with vehicle or P4 (100 nM) for 2 h before subjecting to fixation and PLA analysis. Scale bar, 40 μm. (**m**) Cytoplasmic (C) and nuclear (N) fractionation analysis of PRA and PRB following stimulation with P4 for 15–240 min demonstrates gradual accumulation of PRB in the nucleus and PRA in the cytoplasm. Representative western blots and fractionation controls (Tubulin and Fibrillarin) are shown.

To validate the *in vivo* P4–PR interactions, we used human myometrial cells (hTERT-HM^A/B^) expressing doxycycline (DOX)-inducible PRA and RheoSwitch ligand (RSL)-inducible PRB^32^ (expression validation shown in Supplementary Fig. 6), and performed *in situ* PLA in the presence or the absence of P4. These *in vitro* assays revealed a distinct intracellular localization of P4-liganded PRA and PRB: P4-liganded PRA was found in the cytoplasm but was absent in the nuclei (Fig. 5b), whereas P4-liganded PRB was strictly localized in the nucleus (Fig. 5h). In the unliganded state, PRA was primarily localized in the nucleus (Fig. 5c), whereas PRB was found to be exclusively cytoplasmic in localization (Fig. 5i,k). Furthermore, western blot of cytoplasmic and nuclear fractions showed that P4 treatment resulted in the translocation of PRB to the nucleus, while PRA remained predominantly within the cytoplasm (Fig. 5m, Supplementary Fig. 7). Since PRs are unliganded during labour (Fig. 3a,b), we suggest that PRA accounts for the majority of unliganded nuclear PRs in the labouring myometrium.

### PR is unliganded during preterm labour

**Figure 6:**
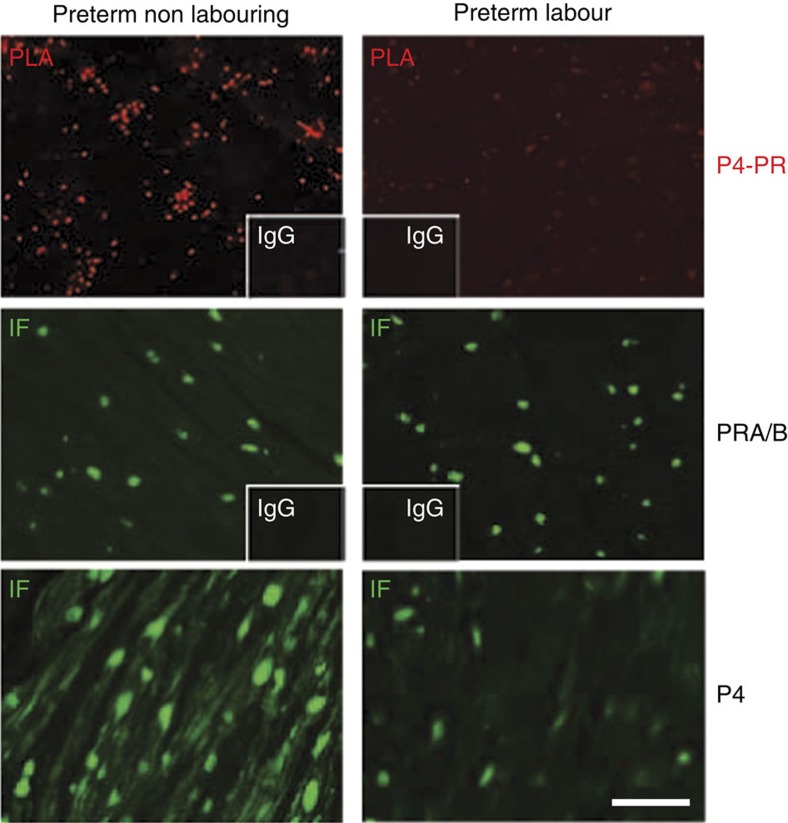
Myometrial PRs are unliganded during preterm labour. Human myometrium from preterm control (non-labouring) and preterm labour. Representative pictures (*n*=3) of: *in situ* Proximity Ligation Assay (PLA) between PR and P4 where red signal represents P4-liganded PRs, and immunofluorescence (IF) with green signal represents total PRs (PRA/B) and P4. Scale bar, 40 μm.

Finally, given the importance of preterm labour as a major driver of perinatal mortality and morbidity in newborns, we examined whether the mechanisms responsible for P4 withdrawal during preterm labour were similar to that of term labour. Myometrial biopsies were collected at emergency C-section from women in active preterm labour and from a pregnancy undergoing second trimester C-section not-in-labour. Importantly, PLA analysis revealed myometrial PRs were unliganded during preterm labour (Fig. 6), whereas the PRs in the preterm myometrium not-in-labour were liganded. Moreover, while there was abundant myometrial nuclear PRs during preterm labour, there was a reduction in intracellular P4 as detected by immunofluorescence (Fig. 6). These data suggest that the reduced intracellular P4, the dissociation of PRs from P4 and the presence of unliganded PRs within the nucleus represents a common element of both term and preterm human labour.

## Discussion

In this study we have identified a mechanism by which the pregnancy-maintaining action of P4 is withdrawn in association with the onset of human labour, even in the presence of elevated circulating levels of this hormone. We show that at the time of human labour PR (predominantly PRA) dissociates from P4 and, therefore, becomes unliganded. We also show that the unliganded PRA localizes to the nucleus where, rather than acting as a repressor of *Cx43* gene transcription, it paradoxically activates transcription of this key labour gene, through mechanisms involving PRA interactions with AP-1 heterodimers. Thus, our data provide a mechanistic explanation of Csapo's ‘functional progesterone withdrawal' theory some 50 years after he postulated how labour could occur in women despite the maintenance of elevated levels of circulating progesterone.

Our data indicate marked differences by which the two PR isoforms, PRA and PRB modulate gene transcription in the pregnant myometrium. During pregnancy under the influence of P4, PRB forms a complex with transcriptional repressors p54^nrb^/mSIN3A/HDAC and inhibits expression of Cx43 (ref. 28). During human labour, we have reported that there is an increase in the ratio of PRA/PRB and suggested that PRA acts to antagonize the repressive functions of PRB on labour gene expression^6^. Here we suggest that it is the unliganded status of PRA that determines its properties as a transcriptional activator. Only in its unliganded state can PRA drives Cx43 transcription. Studies in human breast cancer support this assertion in that the PRB isoform is transcriptionally active when bound to P4, while PRA is transcriptionally active in its unliganded state^33^.

The liganded status of the PR isoforms also has a marked effect on their cellular localization. Classically, unliganded steroid receptors are found in the cytoplasm and are transcriptionally inactive, whereas on binding to their ligand the receptors translocate to the nucleus where they regulate gene transcription. Here we show that while PRB follows this canonical model, PRA exhibits a completely different pattern of cellular localization. In the presence of P4, PRA is cytoplasmic but in the unliganded state it is found predominantly in the nucleus where it induces Cx43 transcription. Differential subcellular distribution of PRA and PRB has been previously described for endometrial and breast cancer cells^34^,^35^. In those studies, in the absence of P4, PRB was localized within the cytoplasm and nucleus, but was primarily nuclear in the presence of P4. In contrast, PRA was predominantly nuclear even in the absence of P4. Unfortunately, those previous studies were not able to discriminate between P4-liganded versus unliganded status of the isoforms.

We show that the ability of PR isoforms to modulate transcription of Cx43 is also dependent on the composition of AP-1 dimers on the Cx43 promoter. JUN/JUN homodimers favour the binding of PRB and the interaction of PRB with the transcriptional repressor complex, p54^nrb^/mSIN3A/HDAC. In contrast, FRA2/JUND heterodimers have reduced affinity for PRB, but instead strongly interact with PRA and induce Cx43 transcriptional activity. Nuclear FRA2 and JUND expression is increased (Supplementary Fig. 2) and, therefore, we suggest that during pregnancy a complex including liganded PRB, JUN/JUN and p54^nrb^/mSIN3A/HDAC suppresses transcription of Cx43, while during labour the unliganded PRA–FRA2/JUND complex is responsible for the increased transcription of this labour gene. Importantly, this mechanism may be applicable to other labour-associated genes, including PTGS2, OXTR, OXN, PTGDS and NFKB2 and several pro-inflammatory cytokines, chemokines and extracellular matrix proteins, which are also known to be regulated by AP-1 transcription factors.

The mechanism by which PRA becomes unliganded at the time of labour remains to be determined. However, Runnebaum and Zander^36^ reported that myometrial P4 levels were reduced and levels of its metabolite, 20α-dihydroprogesterone were increased in the human myometrium during labour, while Williams *et al*.^37^ recently reported that labour is associated with increased expression of the P4 metabolizing enzyme 20α HSD. Our finding that the presence of the unliganded PRA occurs in association with increased expression of 20α HSD and reduced nuclear levels of P4 suggests that intracellular metabolism of P4 may lead to its dissociation from its receptor leading to the downstream events that result in labour.

**Figure 7:**
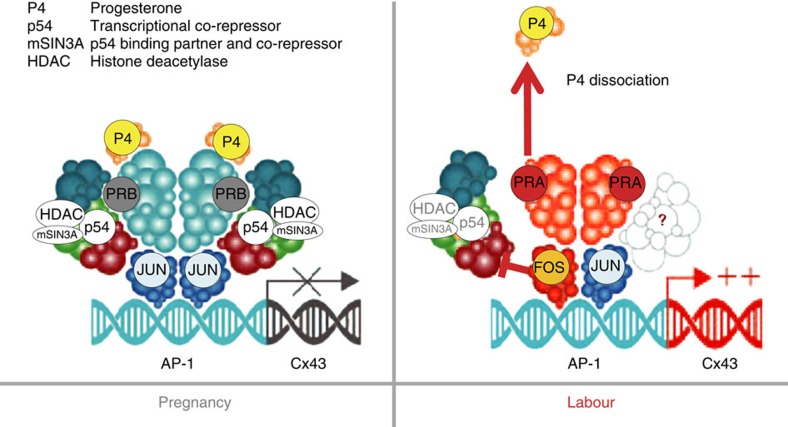
Hypothetical model of Cx43 regulation during pregnancy and term (or preterm) labour. During gestation Jun/Jun dimer complex dominates and interacts with P4-liganded PRs/p54^nrb^/mSIN3A-HDAC transcriptional repressor complex which is recruited to the Cx43 promoter and represses Cx43 transcription. Before the onset of term labour or in the incidence of preterm labour, P4-PR dissociation and simultaneous upregulation of PRA and Fos (FRA2) results in PRA-Fos/Jun heterodimer complex formation (which does not allow incorporation of co-repressor proteins but may facilitate binding of unknown co-activators marked as ‘?'), transcriptional activation of Cx43 and initiation of labour.

In summary, our data represent a paradigm shift in our understanding of the mechanisms by which P4 (through its receptors) controls myometrial contractility during pregnancy and labour. We show that while liganded nuclear PRB can suppress the expression of Cx43, unliganded PRA paradoxically translocates to the nucleus where it acts as a transcriptional activator of this labour gene (Fig. 7). We provide evidence that metabolism of P4 may underlie the reduced nuclear levels of this hormone and its dissociation from its receptor. There has been considerable interest recently in the use of P4 to prevent preterm labour^38^,^39^,^40^, though clinical trials suggest that this therapy is only successful in a subset of patients. Our study raises the possibility that a progestogen that is not subject to metabolism by 20α HSD might represent a more effective therapeutic approach to the prevention of the mortality and morbidity associated with prematurity.

## Methods

### Cell lines and cell culture

Human telomerase immortalized myometrial cells (hTERT-HM) originally developed by Dr Jennifer Condon^30^, were engineered to stably express Flag-PRA or Flag-PRB^29^. Briefly, lentiviral mock vector (backbone: pFUGWBW), Flag-PRA and Flag-PRB expression constructs were transfected into 293T cells along with virapower packaging mix (9 μg, Invitrogen) for 48 h. Lentiviral particles harvested from the conditioned medium of the transfected cells were used to infect hTERT-HM cells. Stable cell lines including hTERT(mock), hTERT(PRA) and hTERT(PRB) were generated after 3 weeks (W) of selection with blasticidin (10 ng μl^−1^). hTERT-HM^A/B^ cell line, with DOX-inducible PRA and RSL-inducible PRB expression, were previously described^32^. Briefly, an hTERT-HM subline was generated by stable transfection of linearized Tet-ON-advanced plasmid and neomycin resistance expression plasmid. Neomycin resistant subline was transfected with linearized pTetON-advanced plasmid with an insert of PRA open reading frame along with linearized DNA for hygromycin resistance. The subline with hygromycin resistance was tested for DOX-mediated induction of PRA expression and further transfected with a RheoSwitch plasmid carrying open reading frame for PRB along with the linearized DNA for basticidin resistance gene and finally the blasticidin resistant subline (hTERT-HM^A/B^) was screened for the DOX induciblity of PRA and RSL inducibility of PRB expression. SHM cells were maintained in DMEM supplemented with 10% FBS, 100 IU ml^−1^ penicillin and 100 μg ml^−1^ streptomycin. Media for hTERT-HM stable cells were further supplemented with the resistance antibiotic; blasticidin 5 μg ml^−1^ and that of hTERT-HM^A/B^ was supplemented with 100 μg ml^−1^ of Geneticin, 1 μg ml^−1^ of Hygromycin B and 5 μg ml^−1^ Blasticidin. All the reagents for cell culture were purchased from Invitrogen Canada, Inc. All the cell lines used in this study were mycoplasma free as assessed by DAPI–DNA staining.

### *In situ* proximity ligation assays

Protein–protein interactions were detected using the Duolink II *in situ* Proximity Ligation Assays (PLA) Detection Kit (Olink Bioscience, Sweden). Briefly, the hTERT-HM PRA and PRB stable cells were cultured on 16-well chamber slides (Lab Tek, Fisher, CA), serum starved for 24 h in serum-free medium and then stimulated with Progesterone (P4, 100 nM) for 2 h. Cells were then washed with cold PBS and fixed with cold methanol: acetone (1:1) for 3 min. After washing, cells were permeabilized with 0.2% Triton X-100 for 5 min, blocked with Olink blocking solution for 1 h and incubated with primary antibodies for overnight at 4 °C. Selection of primary antibodies from different species sources was carefully performed and chosen antibodies presented negligible background. Antibodies from the same secondary source were pre-conjugated with probes to avoid cross reactions. Hybridization with PLA probes (plus and minus), ligation and amplification of conjugants was performed as per manufacturer's instructions. Stringent negative controls were included in all experiments. Slides were mounted with DAPI containing anti-fade mounting medium (Olink, Bioscience) and pictures were taken at constant exposure and × 200 magnification by Leica DM IL LED-Inverted fluorescence microscope with micropublisher 5.0 RTV Q imaging system. Images were analysed by Duolink Image analysis software according to the instructions.

### Collection of murine gestational myometrial tissues

Hsd:ICR (CD-1) outbred mice were mated and vaginal plug detection day was considered day 0.5 of pregnancy. Animals were killed and myometrial samples were collected on gestational day (GD) 15 (non-labouring) and during term labour (GD19). Tissue was collected at 10:00 hours (*n*=3 or more/GD) with the exception of GD19 sample that was collected during active labour as was reported previously^11^. Uterine horns were bisected longitudinally and dissected away from pups and placentas in ice-cold PBS. The decidua basalis and decidua parietalis were removed from the myometrial tissue by cutting/mechanical scraping on ice, respectively, as described earlier^41^. The myometrial tissue was frozen in liquid nitrogen and stored at −80 °C. All mice were housed under specific pathogen-free conditions at the Toronto Centre for Phenogenomics and all animal experiments were approved by the Toronto Centre for Phenogenomics Animal Care Committee.

### Collection of human non-labouring and labouring myometrial tissues

The study design was approved by the Institutional Research Ethics Board of Mount Sinai Hospital, Toronto. Healthy pregnant women with a singleton pregnancy undergoing elective caesarean delivery at term (gestational age ≥37W) were recruited for ‘non-labouring' myometrial tissue collection (*n*=8). Caesarean delivery of ‘labouring' (*n*=6) patients was performed after the onset of labour (regular uterine contractions every 10 min, cervical dilatation >3 cm). Similarly, tissue biopsies were collected from women undergoing emergency C-section in active preterm labour (*n*=3, one case of chorioamnionitis (28W) and two idiopathic preterms (29+7W, 34W) and from pregnancies undergoing second trimester C-section not-in-labour (*n*=3, 32W, 31+6W, 29+4W). A written consent to participate in the study was obtained from each patient. The myometrial biopsy sample (∼1 cm^3^) was excised from the upper margin of an incision made in the lower uterine segment after delivery of the fetus. The biopsy was washed in ice-cold PBS and immediately placed in 10% neutral-buffered formalin (Harleco, Baltimore, MD) or 4% paraformaldehyde (Electron Microscopy Sciences, Hartfield, PA) for fixation. Samples were fixed for 24 h at 4 °C.

### Quantification of P4 levels in myometrial tissue by ELISA

P4 ELISA was conducted on the cytoplasmic and nuclear tissue lysates were prepared from non-labouring and labouring human myometrium (*n*=11 each), using DRG Progesterone Enzyme Immunoassay Kit, DRG, Germany) as per manufacturer's instructions. Statistical analysis was performed using *t*-test for equality of variance.

### Immunohistochemical staining of human myometrial tissue

Immunohistochemistry and immunofluorescence experiments were performed using standard procedures as previously described^41^,^42^. Antibodies used in immunohistochemistry and immunofluorescence are listed in Supplementary Table 1.

### Protein extraction and immunoblotting

Total cell lysates were prepared in lysis buffer (0.08 M Tris/HCl (pH 6.8), 2% SDS, 10% Glycerol) with freshly added protease and phosphatase inhibitor cocktail (Thermo Fisher Scientific, Inc.). Cytoplasmic and nuclear lysates from tissues were prepared with NE-PER, Nuclear and Cytoplasmic Extraction kit (Pierce, USA). Equal amount of protein was separated by SDS–PAGE and transferred to a polyvinylidene difluoride membrane (Trans-blot Turbo Midi PVDF, Bio-Rad) using Turbo Trans-Blot system (Bio-Rad). After blocking for an hour with 5% milk in TBS-T, the membranes were incubated with primary antibody at 4 °C for overnight. The membranes were subsequently probed with horseradish peroxidase-conjugated secondary antibody at room temperature for 1 h. Signals were detected using Luminata HRP-substrate (Millipore) and imaging was performed with VersaDoc imaging system (Bio-Rad). Antibodies used for immunoblotting are listed in Supplementary Table 1. α-tubulin (Sigma-Aldrich, USA), was used as loading control for whole cell lysate and fractionation control for cytosolic proteins, whereas Fibrillarin (Cell Signalling, USA), were used as fractionation controls for nuclear proteins. Secondary antibodies for rabbit, mouse, and goat were purchased from Amersham (dilution range; 1:5,000–10,000) and normal IgGs from Santa Cruz. Densitometric analysis was performed using Image Lab system (Bio-Rad, USA). All uncropped western blots can be found in Supplementary Fig. 7.

### Transient transfection

Transient transfection was performed using jetPRIME (Polyplus) following manufacturer's suggested procedures. The PR expression construct pSG5-PRA and pSG5-PRB, Jun and Fos constructs (pcDNA3.1-cJun, pcDNA3.1- JunB, pcDNA3.1- JunD, pcDNA3.1- cFos, pcDNA3.1- FosB, pcDNA3.1- Fra1 and pcDNA3.1- Fra2), and Cx43 Luciferase promoter constructs (pCx1686-luc, pCx300-luc, and pCx300(m)-luc) were described earlier^26^,^43^.

### Co-Immunoprecipitation

Cells were washed twice with ice-cold PBS and lysed with buffer containing 25mMTris-HCl (pH 7.4), 150 mM NaCl, 1 mM EDTA, 1% NP-40, 5% Glycerol and freshly added protease & phosphatase inhibitor cocktail (Thermo Fisher Scientific Inc. 1:100). Protein samples (500–1000 μg) were pre-cleared with washed protein A or G conjugated agarose beads for 2 h at 4 °C and the supernatant was incubated with 2–4 μg antibody/IgG control, overnight at 4 °C and then applied to pre-washed Agrose beads 2 h at 4 °C. Agarose beads were then washed three times with Lysis buffer and immunoprecipitates were collected by boiling beads in 40–50 μl of 2 × Laemmli buffer for 10 min. Finally, the supernatant and 10% input was subjected to SDS–PAGE and western blot analysis.

### Luciferase reporter assay

SHM cells were transfected with PRA or PRB, different combinations of AP-1 expression vectors (1:1), luciferase reporter for Cx43 promoter (pCx300-luc, pCx300(m)-luc or pCx1680-luc), and pRSVβgal vector (containing *Escherichia coli lacZ* gene under the Rous sarcoma virus promoter). Cells were recovered 5 h after transfection and treated with P4 or its vehicle for 24 h and then collected in passive lysis buffer (Promega, Madison, WI, USA). Luciferase activity was determined using luciferin reagent from Promega. Transfection efficiency was normalized with β-galactosidase activity. All the experiments were performed in triplicates and repeated at least thrice.

### Statistical analysis

Differences among several groups were determined by one-way analysis of variance, followed by Dunnets Multiple comparison test using *Prism* software (GraphPad Prism; San Diego, CA). Two-way analysis of variance and Bonferroni post-tests were used to compare different variables.

## Additional information

**How to cite this article:** Nadeem, L. *et al*. Molecular evidence of functional progesterone withdrawal in human myometrium. *Nat. Commun.* 7:11565 doi: 10.1038/ncomms11565 (2016).

## Supplementary Material

Supplementary Information Supplementary Figures 1-7 and Supplementary Table 1
