# Supplementary material for: Molecular evidence of functional progesterone withdrawal in human myometrium
Source: Nat Commun. 2016 May 25;7:11565. doi: 10.1038/ncomms11565 (PMC4894948; doi:10.1038/ncomms11565)
Supplement: Supplementary Information — Supplementary Figures 1-7 and Supplementary Table 1 [file ncomms11565-s1.pdf]

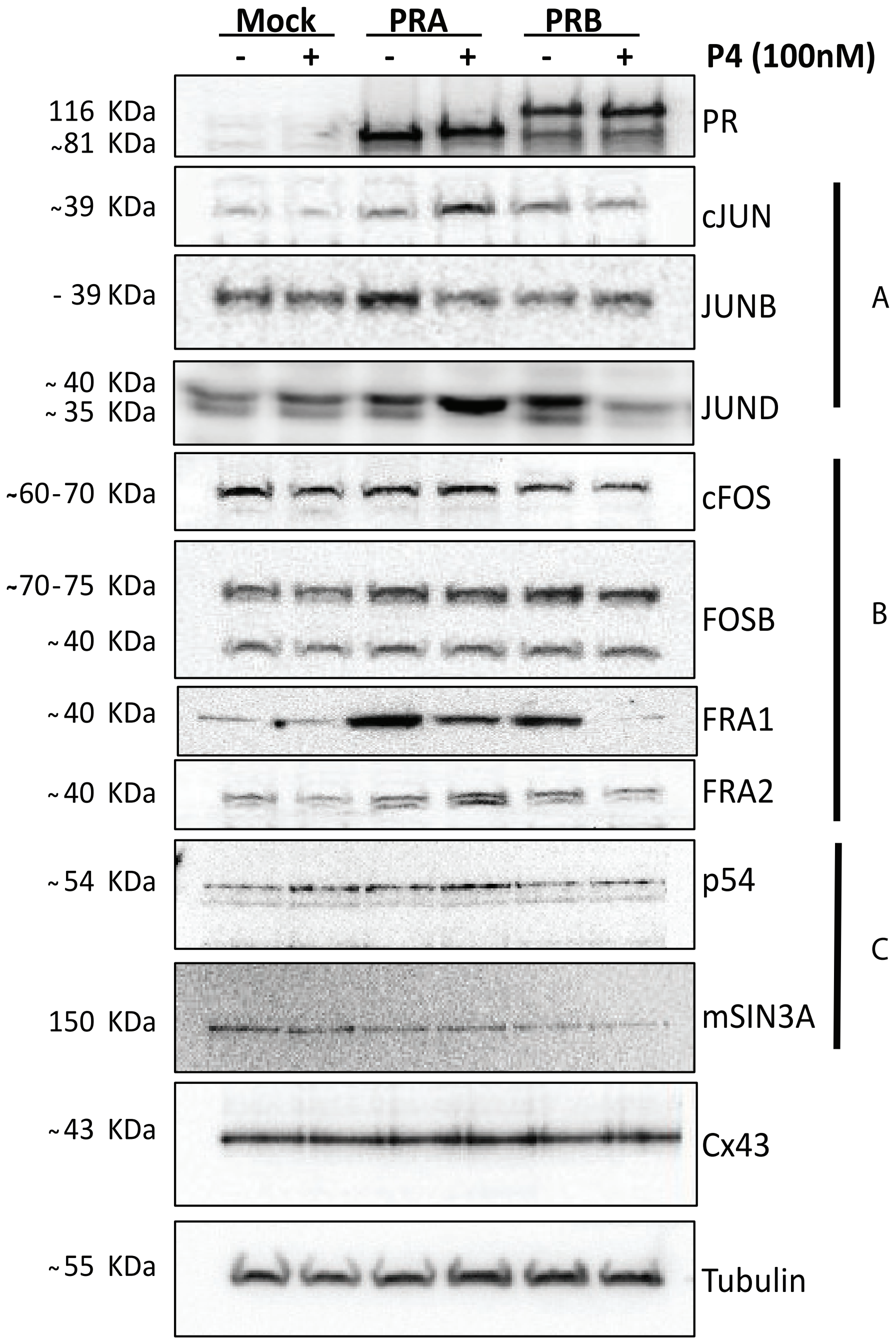

Supplementary Figure 1: **Expression validation of Mock, PRA and PRB stable cell lines .** Western blot analysis of PRA, PRB, endogenous Juns (A), Fos (B), co-repressor proteins (C) and Cx43 in hTERT-HM stable cell lines developed in our laboratory.

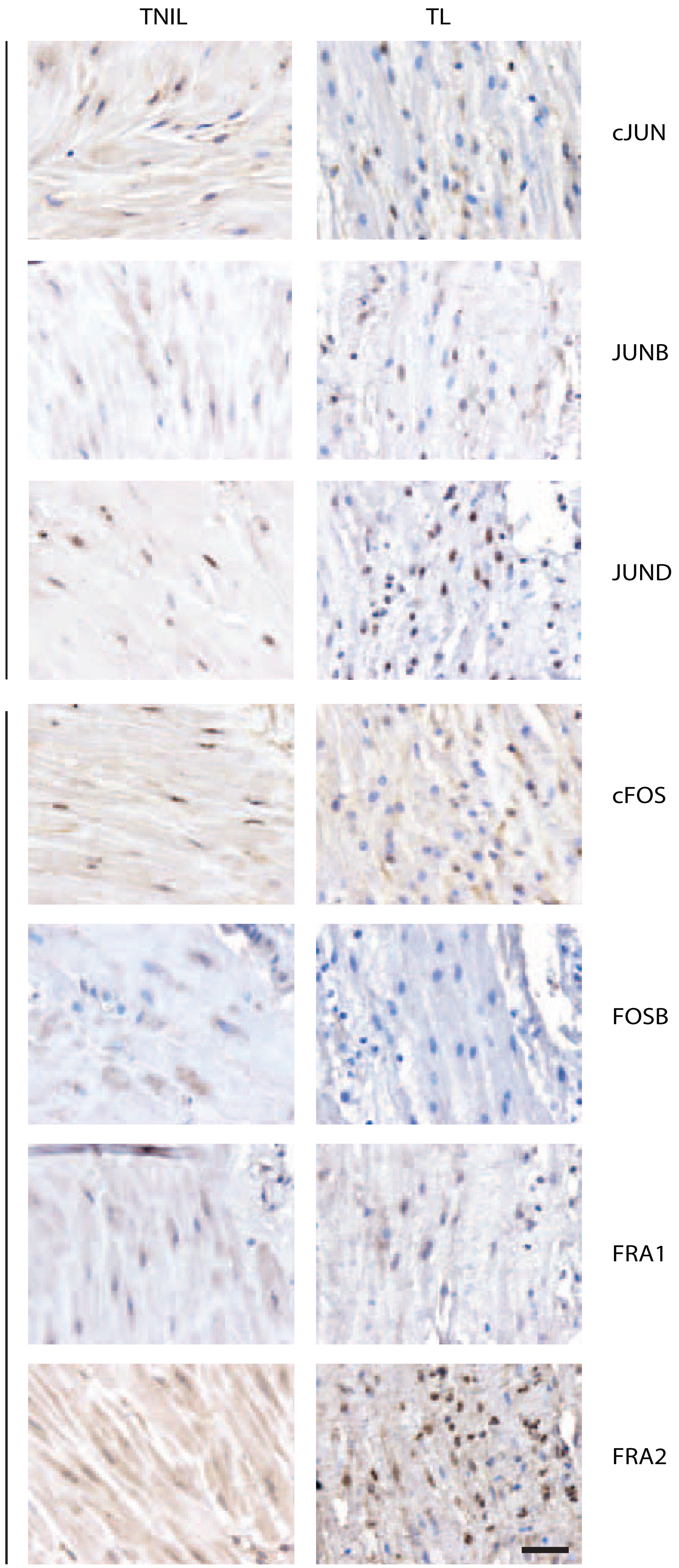

Supplementary Figure 2: Expression of AP-1 factors in human myometrium. Term human myometrium from non-laboring and laboring state. Representative pictures of IHC for JUN and FOS proteins. Scale bar = 40 µm.

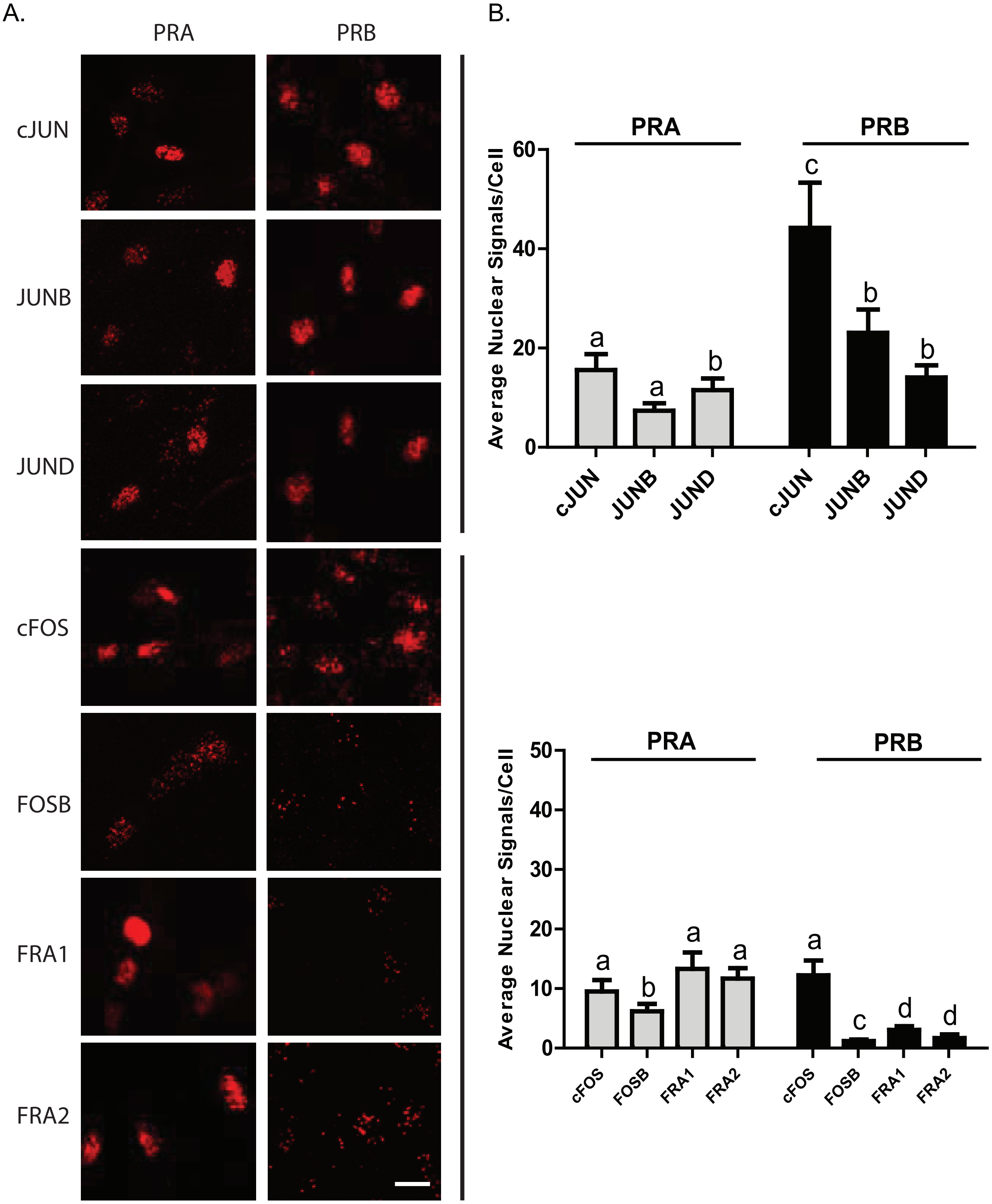

Supplementary Figure 3: Interaction between PRs and AP-1 proteins is P4 independent. In-situ proximity ligation assay (PLA) of PRs with Juns and Fos proteins. A) Representative pics, and B) signal analysis (average nuclear signals/cell from three fields). PRA and PRB stale transfected hTERT-HM cells were treated with vehicle for 2 h and then subjected to PLA analysis. Data represents mean ± SD. n = 3 experiments. Two way ANOVA followed by bonferroni posttests show significant differences between PRA and PRB in the interaction of cJUN (p<0. 001), Fra1 (p<0.001) and Fra2 (p<0.01). Scale bar = 40 µm.

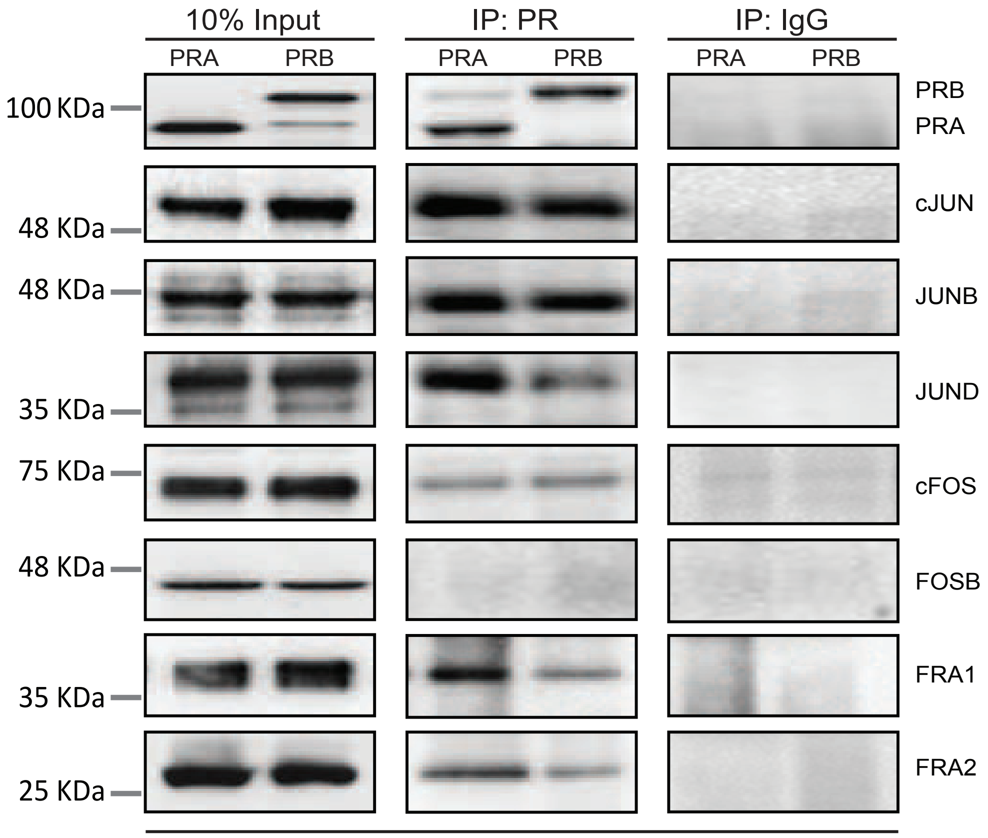

Supplementary Figure 4: **Co-IP analysis confirms the differential associations between PR isoforms and Jun/Fos proteins**. SHM cells were transfected with PRA or PRB along with Jun or Fos expression vectors and treated with 100 nM P4. 250 µg of nuclear lysates were then subjected to Co-IP with PR antibody, immune complex was resolved by SDS-PAGE and immunoblotting with respective Jun or Fos antibodies. 10% Input from the cell lysates is shown in the left panel. Shown are representative blots from two independent experiments.

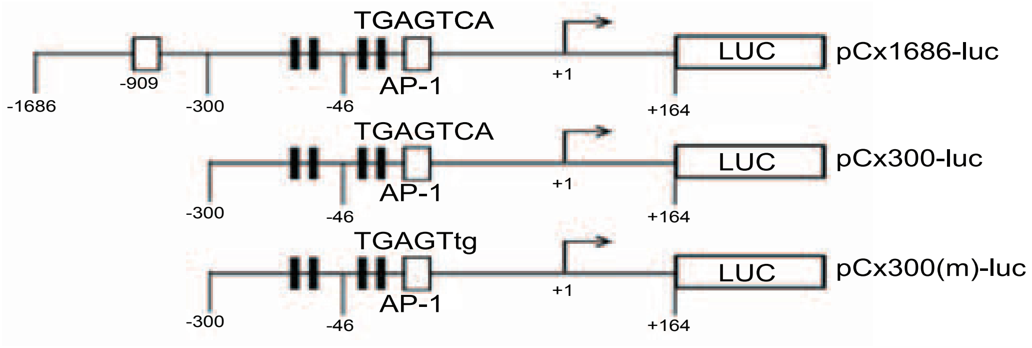

Supplementary Figure 5: Diagram of Cx43 Luciferase reporter vectors used in this study.

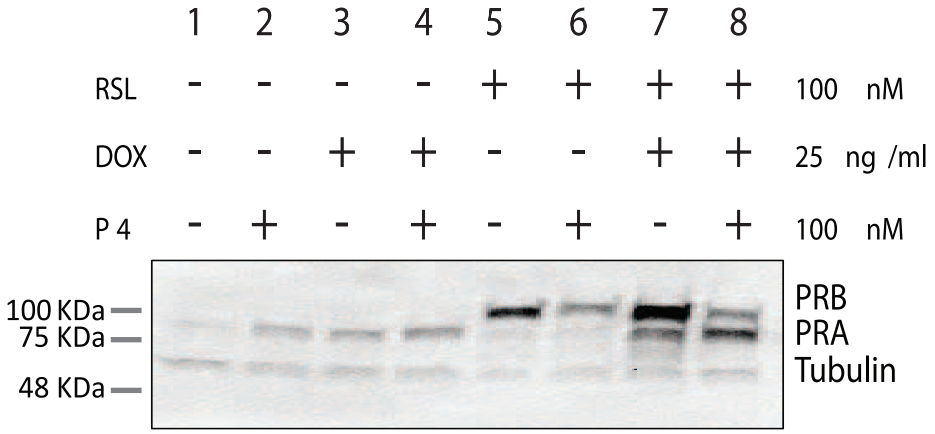

Supplementary Figure 6: Validation of PRA and PRB expression in hTERT-HM$^{A/B}$ cell line induced with either DOX (25 ng/ml) or RSL (100 nM) or both for 24 h and stimulated with vehicle or P4 (100 nM) for additional 2 h. Total lysates were subjected to SDS-PAGE and immunoblotting. Tubulin was used as a loading control.

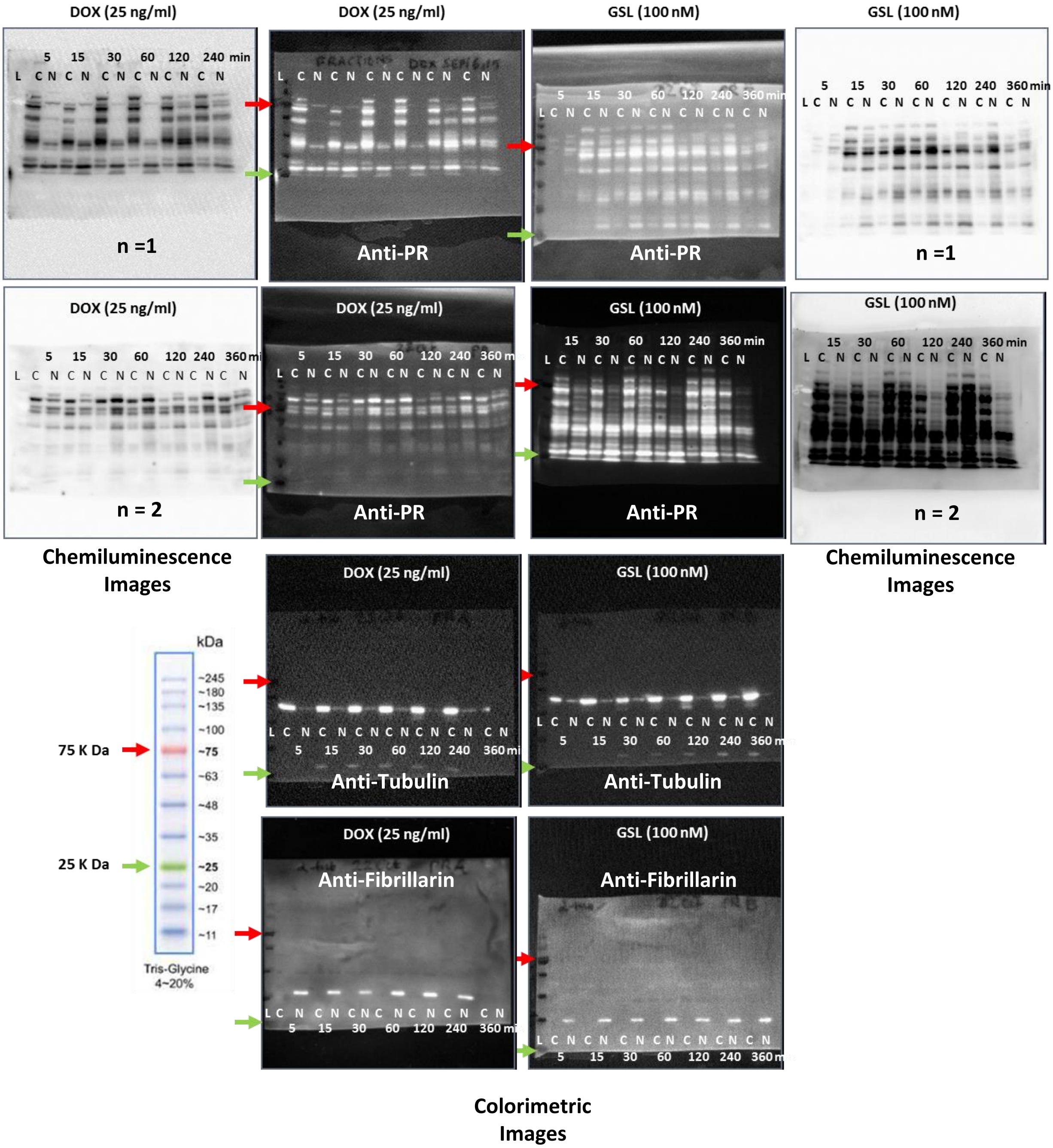

Supplementary Figure 7 A: Uncropped blots and replicates of Figure 5M.

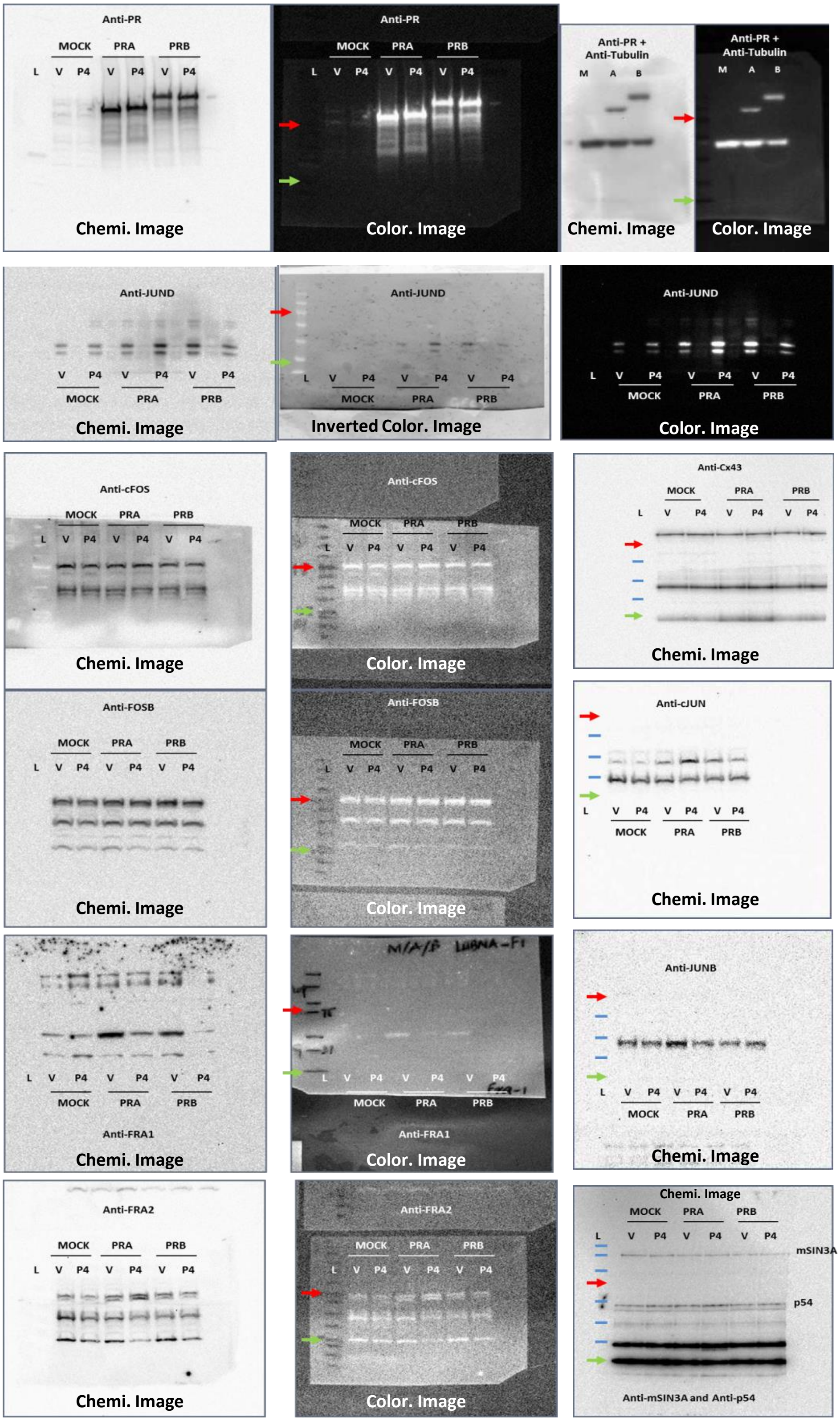

Supplementary Figure 7 B: Uncropped blots or replicates of Supplementary Figure 1.

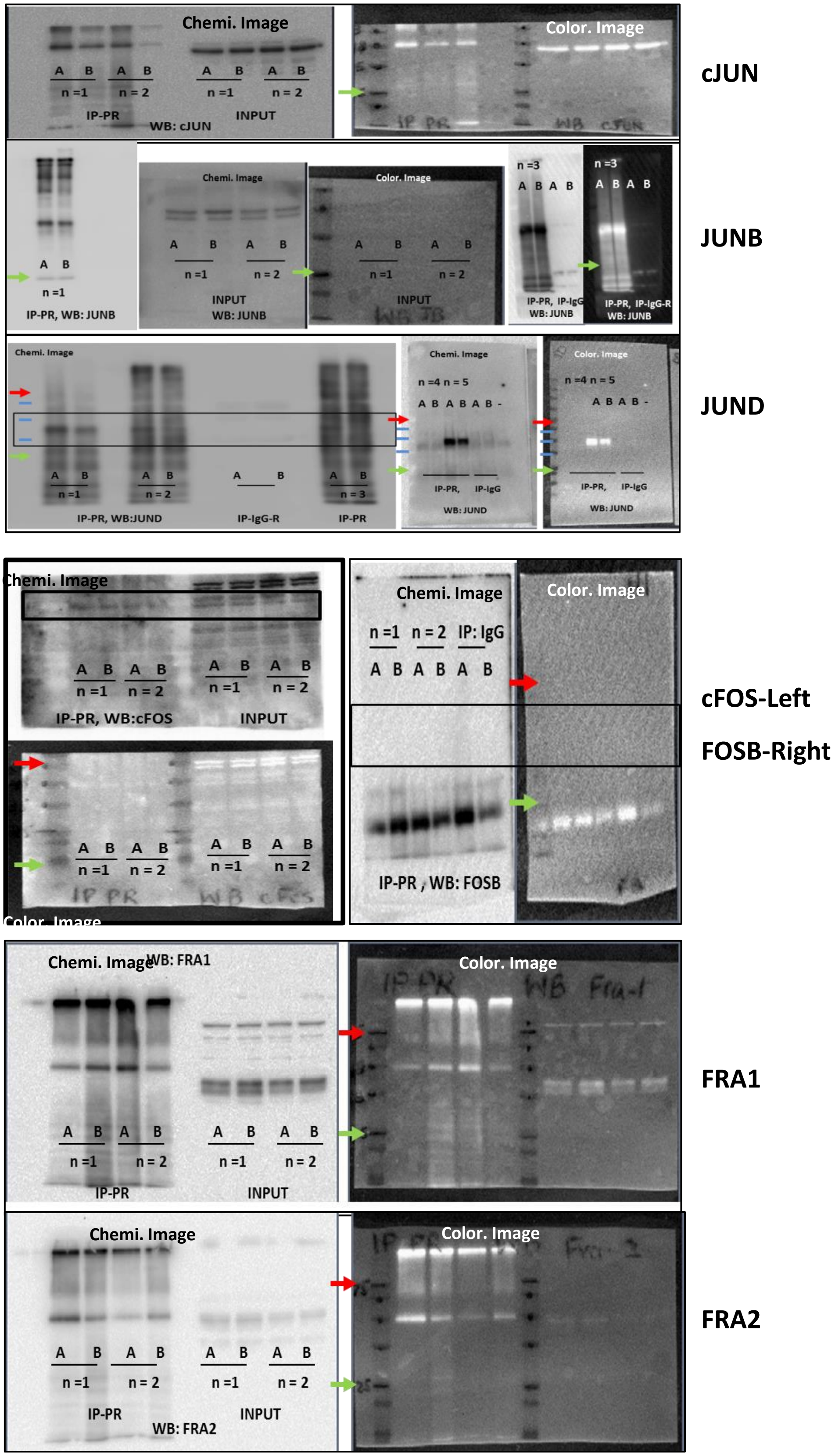

Supplementary Figure 7 C: Uncropped blots or replicates of Supplementary Figure 4.

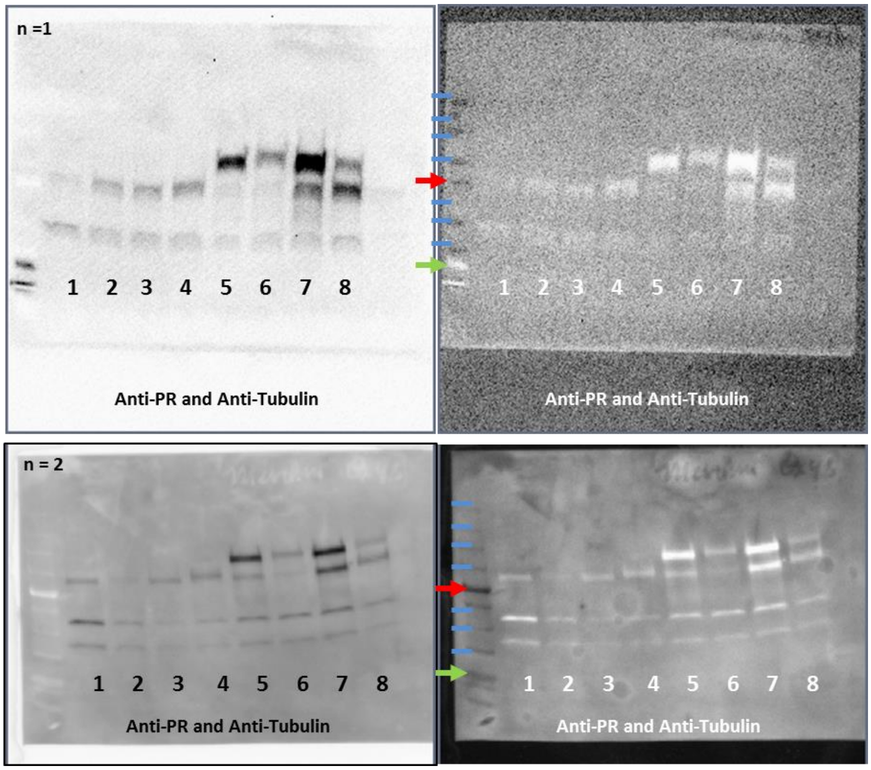

Supplementary Figure 7. D: Uncropped blots and replicate of Supplementary Figure 6.

Supplementary Table 1: Sources and working concentrations of antibodies used in this study

ANTIGEN	ANTIBODY	WORKING CONCENTRATION μg/ml	SOURCE
cJUN	sc-376488	0.2	Santa Cruz
JUNB	sc-73	0.4	Santa Cruz
JUND	sc-74	0.4	Santa Cruz
cFOS	sc-52x	0.4	Santa Cruz
FOSB	sc-52926	0.2	Santa Cruz
FRA1	sc-271657	0.8	Santa Cruz
FRA2	sc-166102	0.4	Santa Cruz
$p54^{nrb}$	sc-166704	0.4	Santa Cruz
mSIN3A	sc-994	0.8	Santa Cruz
Cx43	AB1728	0.2	Millipore
PR	sc-7208	0.2	Santa Cruz
PRB	C1A2	0.2	Cell Sig.
P4	7720-0496	10	AbD Serotec
20α HSD (AKR1C1)	ab183078	5	Abcam
Fibrillarin	2639S	0.015	Cell Sig.
Tubulin	T5168	5	Sigma Aldrich
